# Systematic review and meta-analysis of tube thoracostomy following traumatic chest injury; suction versus water seal

**DOI:** 10.1007/s00068-018-0942-7

**Published:** 2018-03-15

**Authors:** Tim Michael Feenstra, Chris Dickhoff, Jaap Deunk

**Affiliations:** 1 Department of Trauma Surgery, VU University Medical Center, De Boelelaan 1117, 1081 HV Amsterdam, The Netherlands; 2 Department of Thoracic Surgery, VU University Medical Center, De Boelelaan 1117, 1081 HV Amsterdam, The Netherlands

**Keywords:** Trauma, Tube thoracostomy, Chest tube, Suction, Water seal

## Abstract

**Purpose:**

Tube thoracostomy is frequently used in thoracic trauma patients. However, there is no consensus on whether low pressure suction or water seal is the optimal method of tube management. Against this background, we performed a systematic review of studies comparing suction and water seal management of chest tubes placed for traumatic chest injuries in adults. Evaluated outcomes are duration of chest tube treatment, length of stay in hospital, incidence of persistent air leak, clotted hemothorax, and the need for (re-)interventions.

**Methods:**

A systematic literature search according to Preferred Reporting Items for Systematic Reviews and Meta-Analysis (PRISMA) guidelines was performed. Included studies were evaluated according to the Cochrane Collaboration’s tool for assessing the risk of bias, and according to Grading of Recommendations Assessment, Development and Evaluation (GRADE) guidelines for assessing the quality of evidence.

**Results:**

After assessment of 120 identified studies, three RCT’s (randomized controlled trials) were included in this review and meta-analysis. A favorable effect of suction was found for duration of chest tube treatment [MD (mean difference) − 3.38 days, *P* = 0.005], length of stay in hospital (MD −3.90 days, *P* = 0.0003), and the incidence of persistent air leak [OR (odds ratio) 0.27, *P* = 0.001]. No significant difference was found for the incidence of clotted hemothorax and (re-)interventions. The quality of evidence according to GRADE was low, except for persistent air leak (moderate).

**Conclusions:**

Suction seems to have a positive effect on duration of chest tube treatment, length of stay in hospital and persistent air leakage in chest trauma. However, available data was limited and the quality of evidence was (very) low to moderate according to GRADE.

## Background

Thoracic trauma is one of the leading causes of injury-related mortality worldwide, although large geographical variations exist. In the United states of America, chest trauma is responsible for up to 25% of trauma-related deaths, of which up to 20% is caused by penetrating injuries such as stabbing and gunshot wounds [1–3]. European research shows an 18.7% mortality in thoracic trauma cases, with an incidence of blunt trauma of 90% [4]. Mortality differs highly when stratified for injury severity, varying from 5 to 60% mortality when blunt high-impact trauma was studied [5, 6]. Thoracic trauma may lead to fractures of the ribs, disruption of the pleura, injury of other intrathoracic structures, and can be accompanied by concomitant brain- and abdominal trauma. Often a pneumothorax, hemothorax or a combined hemopneumothorax is part of the injury morbidity, which may require a tube thoracostomy for air and fluid evacuation to improve ventilation and cardiac function.

There is a broadly supported agreement amongst surgeons and emergency physicians on the indications and insertion technique of chest tubes, and the procedure is deemed essential according to the Advanced Trauma Life Support (ATLS) and European Trauma Course (ETC) principles [7, 8]. However, tube management after insertion in trauma patients is still subject to considerable debate, particularly on whether water seal or low pressure suction is preferred to achieve full lung expansion and to restore normal function.

Multiple studies have been performed concerning suction versus water seal in postoperative chest tubes, presenting contradictory results [9–17]. Some authors advocate that suction facilitates adequate lung expansion through acceleration of air/fluid evacuation, and therefore, decreases the duration of chest tube treatment [9–11]. Others argue that (mechanical) negative pressure in the pleural cavity harms the pleura and will, therefore, prolong the duration of the air leak, and thus increases the time to chest tube removal [12–15]. Furthermore, since surgical operations and traumatic injuries are different clinical entities, they may need a different approach in tube management. Results from postoperative tube management studies can, therefore, not be extrapolated to trauma patients without caution.

Evaluating duration of chest tube treatment, length of stay in hospital, and complications, the objective of this review was to evaluate which method is superior in tube thoracostomy management after traumatic chest injuries: low pressure suction or water seal.

## Methods

The systematic review and meta-analysis were performed according to the Preferred Reporting Items for Systematic Reviews and Meta-Analysis (PRISMA) guidelines [18].

### Literature search

Two authors (TF and JD) independently conducted a PubMed, Embase, ISI Web of Science, Scopus, ResearchGate and Cochrane Central Register of Controlled Trials and Cochrane Library database extended literature search of all studies published as original articles through July 2017. The literature search was not restricted by date or publication status, and a language restriction was instituted for practical reasons; only English, German and Dutch articles were included. The following terms were used in the search, with all possible synonyms, and in all combinations: “trauma”, “tube thoracostomy”, and “suction” or “water seal”. For instance, in PubMed: (trauma OR injury) AND (“chest tube” OR “tube thoracostomy” OR “chest drain” OR “intrapleural chest drainage” OR “thorax drain”) AND (suction OR “water seal”). To extend the search, the “related article” function was used; the reference lists of articles selected for full-text review were cross-checked for additional articles.

### Study selection

Using piloted forms containing a checklist of the specified inclusion and exclusion criteria, all resulting titles and abstracts were reviewed to determine eligibility. Inclusion criteria were: studies comparing low-pressure suction and water seal management of thoracostomy tubes placed for traumatic chest injuries in adults. This included patients with pneumothoraces, hemothoraces, and hemopneumothoraces. Additionally, studies concerning both blunt and penetrating trauma mechanisms were included. Excluded were studies that did not compare suction and water seal in chest tube management after thoracic trauma. Also excluded were studies that included patients suffering from possible confounding comorbidities; studies examining tube thoracostomy in mechanically ventilated patients, patients suffering from pulmonary or pleural disease, and patients that had a thoracic surgical intervention before or during the placement of the chest tube.

### Risk of bias and methodological quality

The risk of bias of the studies was evaluated using the Cochrane Collaboration’s tool for assessing risk of bias [19]. To objectify the methodological quality of the studies for each outcome parameter, the Grading of Recommendations Assessment, Development and Evaluation (GRADE) guidelines were used [20]. This scoring system awards four points for RCT’s (Randomized Controlled trials) and two points for observational evidence, and retracts points for risk of bias, inconsistency, indirectness and imprecision (smaller size of effect). The total amount of points correlates with the quality of evidence and the strength of the recommendation about the treatment derived thereof; four points correlate with “high quality of evidence”, and therefore, a strong recommendation, three points correlate with “moderate quality of evidence”, and therefore, a moderate strength recommendation, two points correlate with “low quality of evidence”, and therefore, a weak recommendation, one point correlates with “very low quality of evidence”, and therefore, a very weak recommendation. Assessment of the studies was executed by TF and JD, the third author (CD) resolved differences in opinions if needed.

### Primary and secondary study outcomes

Primary outcomes investigated in this review are the duration of the chest tube treatment and length of stay in hospital (LOS). For the secondary outcomes, the incidence of complications of treatment was analyzed: persistent air leak (leading to partial or no lung expansion), remaining or clotted hemothorax, and surgical (re-)interventions (new tube placement, thoracoscopy and thoracotomy) for undrained pleural collection, clotted hemothorax and empyema.

### Statistical analysis

For the duration of chest tube treatment and LOS, the mean difference (MD) was used as effect size. The method suggested by Hozo et al. [21] was used to calculate mean and standard deviation (SD) for the study by Morales et al. [22], which only reports median and range. The method as proposed by Hozo et al., is a frequently used statistical/mathematical technique in meta-analysis to compare studies that report median and range, with studies that mean and standard deviation. For complications, the effect size was estimated by calculating the odds ratio (OR).

The weights assigned to each study were computed according to the inverse of the variance. For complications, this was done by calculating the pooled ORs of the studies, using the Mantel–Haenszel method for random-effect measurement in meta-analysis [23].

MD and OR values are reported with 95% confidence intervals (CIs), and a *P* value < 0.05 was considered statistically significant. All analyses were performed using RevMan version 5.3 (Copenhagen: The Nordic Cochrane Centre, The Cochrane Collaboration, 2014).

## Results

### Study selection

**Fig. 1:**
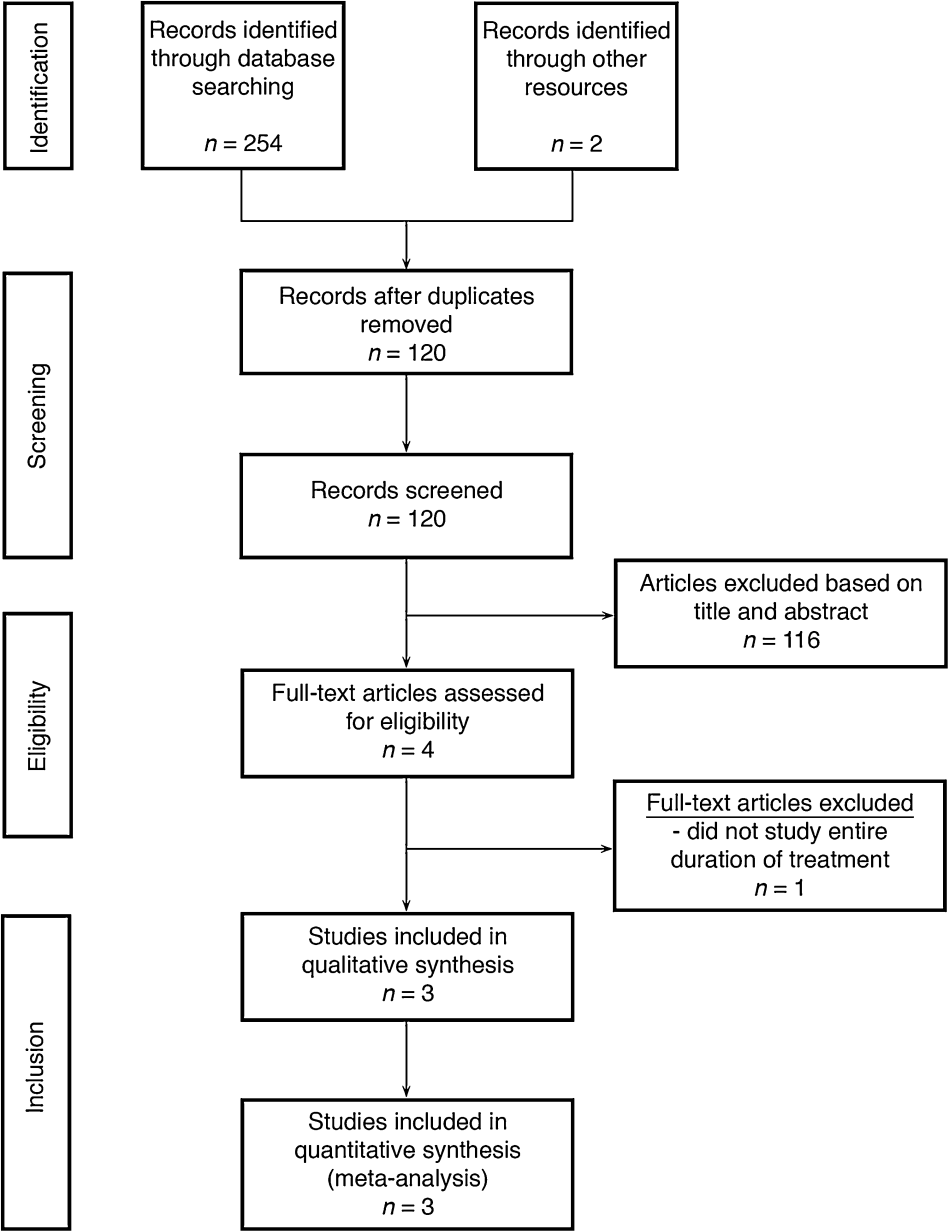
PRISMA flow chart of literature search and article selection

Figure 1 depicts the PRISMA flow chart of the literature search and article selection. 254 studies were identified through database assessments; two studies were identified through cross-checking the reference lists of articles selected for full-text review. After removing duplicates, 120 studies were identified in the literature search. After assessment of title and abstract, 116 articles were excluded based on inclusion and exclusion criteria. The remaining four articles were analyzed full-text for eligibility. One of these four studies was excluded because it compared suction and water seal only during the last few hours before tube removal, instead of during the entire chest tube treatment, and therefore, did not cover our research question. The remaining three studies were included in the review and meta-analysis.

### Study characteristics

The three studies that were finally included in the meta-analysis involved a total of 270 patients (Table 1), 136 (50.4%) were included in the suction group and 134 (49.6%) in the water seal group. 227 patients (84%) were male, 43 (16%) were female. All three studies were prospective randomized trials, comparing water seal and low pressure suction after tube thoracostomy. No retrospective studies comparing the two treatment modalities were found.

**Table 1:** Characteristics of included studies

First author	Year	Total patients	Suction		Water seal		Injury^a^	Indication for tube thoracostomy^b^
			Male (%)	Female (%)	Male (%)	Female (%)		
Majumdar [24]	2014	60	23 (76.7%)	7 (23.3%)	25 (83.3%)	5 (16.7%)	B, P	HT, HPT
Morales [22]	2014	110	54 (96.4%)	2 (3.6%)	51 (94.4%)	3 (5.6%)	B, P	PT, HT, HPT
Muslim [25]	2008	100	38 (76%)	12 (24%)	36 (72%)	14 (18%)	P	Not distinguished
Subtotal			115 (84.6%)	21 (15.4%)	112 (83.6%)	22 (16.1%)		
Total		270	136 (50.4%)		134 (49.6%)			

^a^*B* blunt trauma, *P* penetrating trauma

^b^*PT* pneumothorax, *HT* hemothorax, *HPT* hemopneumothorax

Majumdar et al. [24] included 60 patients with hemothoraces or hemopneumothoraces after both blunt and penetrating chest trauma. Patients were randomized, and appointed to either the intervention (suction) group or the control (water seal) group. Continuous low pressure suction of − 5 to − 20 cm H_2_O was used in the suction group. Endpoints were duration of chest tube treatment, length of stay in hospital, and need for conversion to thoracotomy for clotted hemothorax and empyema. The authors concluded that continuous low pressure suction was favorable over water seal for all endpoints.

Morales et al. [22] included patients suffering from both blunt and penetrating trauma, leading to pneumo-, hemo-, or hemopneumothoraces. 110 patients were randomized, 56 in the suction group, and 54 in the water seal group. A continuous negative pressure of − 20 cm H2O was used in the suction group. Endpoints were the duration of chest tube treatment, LOS and complications. The authors did not find a difference between suction and water seal for all of these endpoints.

Muslim et al. [25] investigated patients after penetrating chest trauma resulting in pneumothoraces, hemothoraces or hemopneumothoraces. 100 patients were randomized and equally allocated in the suction group and in the water seal group. A continuous negative pressure of− 20 cm H_2_O was used in the suction group. Endpoints were duration of chest tube treatment, LOS, persistent pneumothorax, and clotted hemothorax. Suction was favorable for all endpoints, except for clotted hemothorax, for which there was no significant difference in incidence.

### Quality of trials

Table 2 shows the assessment of the risk of bias, agreed on by two authors (TF and JD), of the individual studies using the Cochrane Collaboration’s tool for assessing risk of bias. None of the studies provided adequate blinding of patients, treating physicians, and researchers. This will result in a high risk of bias, especially for the primary outcomes (duration of chest tube treatment and LOS) as these results are more subjective than the secondary outcomes (complications). All studies are randomized, albeit that the randomization of Majumdar (through even or uneven numbering of patients) and Muslim (through distributed envelopes) did not meet Cochrane standards. In addition, Majumdar did not supply a definition or protocol for the indication of chest tube removal or patient discharge, resulting in an increased bias of unknown nature.

**Table 2:** Cochrane Collaboration’s tool for assessing risk of bias

First author	Selection bias		Performance bias	Detection bias	Attrition bias	Reporting bias	Other bias
	Random sequence generation	Allocation concealment	Blinding of participants and personnell	Blinding of outcome assessment	Incomplete outcome data	Selective reporting	
Majumdar [24]	+	−	−	−	+	+	−
Morales [22]	+	+	−	−	+	+	+
Muslim [25]	+	?	−	−	+	+	?

+ low risk of bias, − high risk of bias, ? unknown risk of bias

### Primary outcomes

#### Duration of chest tube treatment

**Fig. 2:**
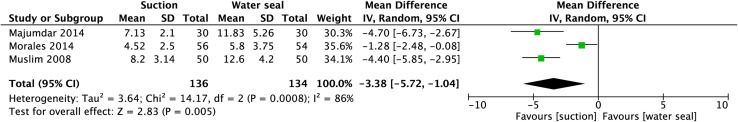
Meta-analysis of duration of chest tube treatment. A Mantel–Haenszel random effects model was used. Mean differences are shown with 95% confidence intervals

Meta-analysis of the duration of chest tube treatment shows a significant favorable effect for suction, with a mean difference of 3.38 days [95% CI (− 5.72, − 1.04), *P* = 0.005] (Fig. 2).

#### Length of stay in hospital (LOS)

**Fig. 3:**
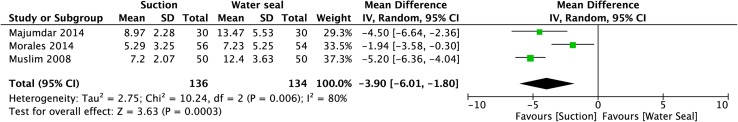
Meta-analysis of duration of length of stay in hospital. A Mantel–Haenszel random effects model was used. Mean differences are shown with 95% confidence interval

Meta-analysis of length of stay in hospital shows a significant favorable effect for suction, with a mean difference of 3.90 days [95% CI (− 6.01, − 1.80), *P* = 0.0003] (Fig. 3).

### Secondary outcomes

#### Persistent air leak

**Fig. 4:**
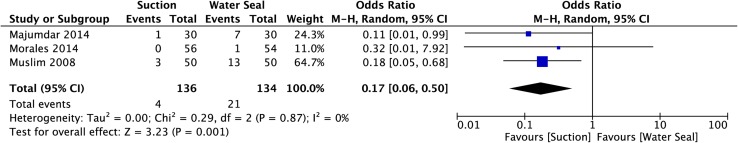
Meta-analysis of incidence of persistent air leak. A Mantel–Haenszel random effects model was used. Odds ratios are shown with 95% confidence intervals

Meta-analysis of persistent air leak shows a significant favorable effect for suction [OR 0.27, 95% CI (0.06, 0.50), *P* = 0.001] (Fig. 4).

#### Clotted hemothorax

**Fig. 5:**
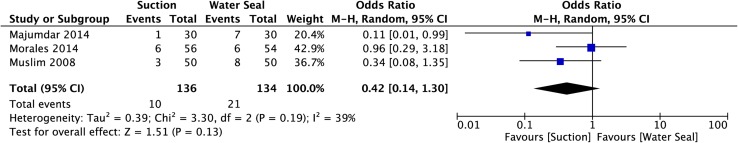
Meta-analysis of incidence of persistent clotted hemothorax. A Mantel–Haenszel random effects model was used. Odds ratios are shown with 95% confidence intervals

Meta-analysis of clotted hemothorax does not show a significant favorable effect for either treatment [OR 0.42, 95% CI (0.14, 1.30), *P* = 0.13] (Fig. 5).

#### (Re-)interventions

**Fig. 6:**
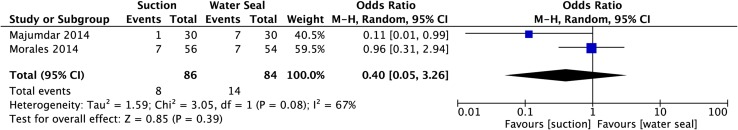
Meta-analysis of incidence of (re-)interventions. A Mantel–Haenszel random effects model was used. Odds ratios are shown with 95% confidence interval

Only Majumdar and Morales included (re-)interventions (new tube placement, thoracoscopy and thoracotomy) as outcomes. Meta-analysis of these (re-)interventions does not show a significant favorable effect for either treatment [OR 0.40 (0.05, 3.26), *P* = 0.39] (Fig. 6).

### GRADE

Table 3 shows the GRADE scores for the quality of the evidence for each outcome parameter in the available studies. For the outcomes duration of chest tube management and length of stay in hospital, the quality of evidence was scored as “very low quality” because of risk of bias, inconsistency and indirectness. For the outcome persistent air leak, the quality of evidence was scored as “moderate quality” because of indirectness. For the outcome clotted hemothorax, the quality of evidence was scored as “low quality” because of indirectness and imprecision. For the outcome (re-)interventions the quality of evidence was scored as “very low quality” because of inconsistency, indirectness and imprecision.

**Table 3:** Summary of findings and GRADE quality of evidence score

Outcomes	Anticipated absolute effects^a^ (95% CI)		Relative effect (95% CI)	No. of participants (studies)	Quality of evidence (GRADE)
	Risk with water seal	Risk with suction			
Duration of chest tube treatment	The mean duration of the chest tube treatment was 9.95 days	The mean duration of the chest tube treatment in the suction group was 3.38 days shorter (5.72–1.04 shorter)	–	270 (3 RCT’s)	Very low
Length of stay in hospital (LOS)	The mean length of stay in hospital was 10.99 days	The mean length of stay in hospital in the suction group was 3.9 days shorter (6.01–1.8 shorter)	–	270 (3 RCT’s)	Very low
Persistent air leak	157 per 1.000	31 per 1.000 (11–85)	OR 0.17 (0.06–0.50)	270 (3 RCT’s)	Moderate
Clotted hemothorax	157 per 1.000	72 per 1.000 (25–195)	OR 0.42 (0.14–1.30)	270 (3 RCT’s)	Low
(Re-)interventions	155 per 1.000	68 per 1.000 (9–373)	OR 0.40 (0.05–3.25)	170 (2 RCT’s)	Very low

*CI* confidence interval, *MD* mean difference, *OR* odds ratio

^a^The risk in the intervention group (and its 95% confidence interval) is based on the assumed risk in the comparison group and the relative effect of the intervention (and its 95% CI)

## Discussion

This systematic review and meta-analysis aims to combine all available studies comparing low pressure suction and water seal management of chest tubes in trauma patients. However, although tube thoracostomy is a frequently executed procedure following thoracic trauma, very little evidence exists concerning the preferred tube pressure management (low pressure suction or water seal) after tube placement. Three relevant studies met inclusion criteria and could, therefore, be included in this review. The results of this review indicate that there is some evidence that suction is the favorable management of chest tubes in the treatment of traumatic pneumo-, hemo-, and hemopneumothoraces. The duration of chest tube treatment and the length of stay in hospital were significantly decreased by 3 days, and the incidence of persistent air leak decreased significantly in the suction group. For the incidence of clotted hemothorax and the need for (re-)interventions (new tube placement, thoracoscopy and thoracotomy), the effect of suction was not significant.

Although evidence in trauma cases for the preferred management of chest tubes is scarce, there is ample research in the field of postoperative chest tube management in lung surgery. Two systematic reviews and meta-analysis on postoperative tube management have been conducted, including comparable RCT’s. Coughlin et al. [26] found no advantage of suction over water seal regarding duration and incidence of (prolonged) air leak, duration of chest tube treatment, and length of hospital stay in 2012. Lang et al., published their review in 2016 and included eight RCT’s that investigated tube management after non-pneumonectomy lung resections. They found that, although suction improves air and fluid removal from the pleural space, it does not improve clinical outcomes such as duration of chest tube treatment and length of stay in hospital after surgery [27].

In this present review on suction versus water seal in traumatic injuries, a favorable effect of suction on most outcomes was found. However, the total number of studied patients was relatively low (*n* = 270) and all included studies had some methodological imperfections leading to a risk of bias. Blinding of patients, physicians and researchers was inadequate for all three included studies leading to an increased risk of performance and detection bias, affecting especially the primary outcomes (duration of chest tube management and length of stay in hospital) because of their relative subjective nature. The lack of a study protocol for the indication of chest tube removal or patient discharge of Majumdar et al. enhances the risk of bias even more, although the exact consequence is unknown and hard to predict.

GRADE analysis revealed that the overall quality of evidence per outcome was moderate, low or even very low (Table 3). All three included studies were considered ‘randomized controlled trials’, and all outcomes, therefore, started as high quality of evidence. However, risk of bias was a problem for the primary outcomes, as mentioned before, leading to the retraction of one point for quality. In addition to that, pneumothoraces, hemothoraces, hemopneumothoraces, and both sharp trauma and blunt trauma were all studied together, which lead to the retraction of one point for indirectness. Moreover, the heterogeneity of the results amongst the studies was high, leading to retraction of one point for inconsistency. The result is a low quality of evidence score for the two primary outcomes. In addition, duration of chest tube treatment and length of stay in hospital may be affected by a range of confounding factors, most notably by differences in trauma mechanism, injury severity, and concomitant injuries. However, these potential confounders are not described in any of the three studies, and therefore, their effect on the outcomes is unknown.

For the GRADE-score of persistent air leak, only one point was retracted for indirectness, meaning the quality of evidence is considered moderate. For clotted hemothorax, the quality of evidence was graded low, one point was extracted for indirectness and one point for imprecision (95% CI includes the limit of clinical relevance). For (re-)interventions the quality of evidence was very low because of inconsistency (heterogeneity between the studies), indirectness, and imprecision (95% CI includes the limit of clinical relevance).

A statistical challenge of the meta-analysis in this study was the comparison of the study by Morales et al. [22] that did not report their results in mean and standard deviation, but in median and range. To facilitate the meta-analysis, the method of Hozo et al. [21] was used. This is a widely accepted and used statistical/mathematical method for this purpose. There have been some remarks that this method does not fully incorporate the sample size and may lead to overestimation. However, as the sample size in this review is (relatively) small, this issue is of less impact and will not alter the conclusions of this study [28, 29].

For complications, the weights assigned to each study were computed by calculating the pooled OR’s. Under the assumption that included studies estimate a different yet related intervention effect on the outcomes, and the assumption of inter-study heterogeneity of a random nature, the Mantel–Haenszel statistical method with random effects model for statistical evaluation was used. Another possible statistical method to compute the weights assigned to each study, could have been the inverse-variance method. These methods calculate the weights in a slightly different way, and differences in results between the two methods are likely to be trivial [23].

None of the studies included in the current review included patients on mechanical ventilation, and little research concerning chest tubes in mechanically ventilated trauma patients exists. However, there is an indication that chest tubes may lead to bronchopleural fistula more frequently in mechanically ventilated patients [30, 31]. More research is highly advised, as the need for mechanical ventilation is very common in severely injured trauma patients.

Suction was continued until drain removal in all three included studies, yet evidence for tube management in the last hours before removal is contradictory. In theory, a short period of water seal or clamping of the tube might reveal an occult air leak or pneumothorax. In 1994 Davis et al. [32] compared continuing suction with a short period of water seal before removal of the chest tube, and found that continuing suction until chest tube removal reduced total chest tube duration and required shorter time to remove the chest tube following air leak resolution. In contrast, Martino et al. and Bridges et al., found no difference between continuing suction and a short water seal in chest tube duration or hospital length of stay between suction or a short water seal before chest tube removal [33, 34]. Because the results from these studies are contradictory, it is hard to give a firm recommendation for the management just before drain removal.

Although all three studies utilize low pressure suction, there is no consensus concerning the amount of negative pressure suction in this review. Morales et al. and Muslim et al. apply the commonly used negative pressure of − 20 cm H_2_O, yet Majumdar et al., apply a negative pressure of − 5 to − 20 cm H_2_O.

Surgical technique of the tube thoracostomy (incision versus percutaneous chest tab) is not reported by two of three studies, and only mentioned by Morales et al. Therefore, it is impossible to draw any conclusion concerning the superiority of either surgical technique.

Another topic that was not studied by this review, is immediate thoracoscopic suction and evacuation of hemothoraces and hemopneumothoraces before tube placement. According to some authors, this intervention may shorten chest tube duration, and decrease the incidence of empyema and recurrent pneumothorax, and the need for additional drainage [35, 36]. However, results of these studies are contradictory and the value of thoracoscopic suction and evacuation still remains debated.

This review was limited by the small amount of available trials comparing—low pressure suction and water seal in the management of chest tubes placed after traumatic chest injuries. Only three studies, with a total number of 270 patients were found in the literature. Moreover, all three studies had some methodological imperfections and the overall quality of evidence for the different outcomes was (very) low.

To improve the overall quality of evidence and to support better founded conclusions, more and larger randomized controlled trials are needed, that research the management of tube thoracostomy for thoracic injury by suction or water seal. These studies should favorably differentiate between pneumo-, hemo-, and hemopneumothoraces, as well as between blunt and penetrating thoracic injuries. In addition, new studies are needed that research chest tubes in mechanically ventilated patients.

## Conclusion

In the management of tube thoracostomy for chest trauma in adults, there seems to be some evidence suggesting that suction decreases duration of chest tube treatment, length of hospital stay, and persistent air leakage. Suction does not seem to have a significant effect on the incidence of clotted hemothorax and the need for (re-)interventions. This review was limited by small amount of studies on this topic, describing only a limited number of patients. Moreover, the quality of the evidence for most studied outcomes, was low according to GRADE. Therefore, conclusions should be interpreted with caution, and more studies are needed to provide stronger evidence and to affirm the conclusions from this review.
